# Mechanistic Insight into the Anti-Bacterial/Anti-Biofilm Effects of Low Chlorhexidine Concentrations on *Enterococcus faecalis*—In Vitro Study

**DOI:** 10.3390/microorganisms12112297

**Published:** 2024-11-12

**Authors:** Nathanyel Sebbane, Itzhak Abramovitz, Nurit Kot-Limon, Doron Steinberg

**Affiliations:** 1 Biofilm Research Laboratory, The Institute of Biomedical and Oral Research (IBOR), The Faculty of Dental Medicine, The Hebrew University of Jerusalem, Jerusalem 9112102, Israel; nathanye.sebbane@mail.huji.ac.il; 2 Department of Endodontics, The Faculty of Dental Medicine, The Hebrew University-Hadassah, Jerusalem 9112102, Israel; itzhakab@hadassah.org.il (I.A.); nurit.kot@mail.huji.ac.il (N.K.-L.); 3 “Bina” Program, Faculty of Dental Medicine, The Hebrew University-Hadassah, Jerusalem 9112102, Israel

**Keywords:** *E. faecalis*, chlorhexidine, intracanal medication, endodontic treatment, biofilm

## Abstract

Background: Endodontic treatment failures are often linked to the persistence of *Enterococcus faecalis* in the root canal system. This study aimed to investigate the antibacterial/antibiofilm mechanism of chlorhexidine (CHX), particularly at low concentrations, against *E. faecalis*, to improve endodontic treatment protocols. Methods: The antibacterial activity of CHX (0.125–20 μg/mL) was evaluated against *E. faecalis* ATCC 29212 using various assays, including planktonic growth inhibition, colony-forming units (CFUs), membrane permeability and potential assays, high-resolution scanning electron microscopy (HR-SEM), confocal laser scanning microscopy of biofilms, biomass and metabolic activity assays on matured biofilm, and quantitative real-time PCR for gene expression. Statistical analysis was performed using Student’s *t*-test and ANOVA. Results: CHX demonstrated concentration-dependent inhibition of *E. faecalis*, significantly reducing planktonic growth and CFUs. Membrane assays showed increased permeability and depolarization, indicating damage. HR-SEM revealed morphological changes, such as pore formation, while confocal microscopy showed a reduction in biofilm mass and extracellular substances. Gene expression analysis indicated the downregulation of virulence genes and upregulation of stress response genes. Conclusions: CHX at low concentrations disrupts *E. faecalis* at multiple levels, from membrane disruption to gene expression modulation, affecting mature biofilm. These findings support the refinement of endodontic disinfection protocols to reduce microbial persistence.

## 1. Introduction

Since the role of bacteria in the etiology of endodontic disease has been established [1], relentless efforts have been invested in finding optimal strategies to minimize or eradicate their presence in the root canal system. Traditionally, these strategies include a combination of chemo-mechanical preparations that combine methods of mechanical debridement supplemented by irrigation with various solutions with antibacterial properties, to enhance disinfection processes [2]. Dressing the root canals with antibacterial materials to obtain a better antibacterial effect was not proven clinically significant and was not addressed in this study. Irrigation involves antiseptic solutions, such as sodium hypochlorite (SHC), chlorhexidine gluconate, and iodine potassium iodide (IKI) in various concentrations, or premixed formulations, such as MTAD (a mixture of doxycycline, citric acid, and a detergent). Though SHC is the most abundant and recommended material for root canal irrigation, its cytotoxicity cannot be overlooked. Accidental infusion of SHC beyond the apical foramen may induce substantial deleterious effect on the organism including vast ecchymosis, renal damage, and body tissue irreversible damage [3].

Bas du formulaire

Biofilms play a critical role in the persistence of root canal infections, while these microbial communities are adept at colonizing dentinal tubules and root canal spaces, necessitating effective intervention [4,5].

Within the spectrum of microbial adversaries, *Enterococcus faecalis*, a Gram-positive bacterium, stands out for its notorious role in the failure of endodontic treatments. Its resilience, demonstrated by its ability to survive endodontic procedures and infiltrate dentinal tubules to depths of up to 800 microns, underscores a critical challenge [5]. It is important to note that the pathogenicity of *E. faecalis* is part of a broader bacterial community within biofilms, involving multiple species [5]. The pathogenicity of *E. faecalis* in endodontic failures calls for targeted antimicrobial strategies, as demonstrated by studies emphasizing the bacterium’s resistance to standard disinfectants and its prevalence in persistent infections [6]. Its ability to form biofilms that structurally protect it and to activate proton pumps that inactivate calcium hydroxide’s antibacterial effect makes it particularly challenging to eradicate, as recently highlighted [7], and its prevalence in failed endodontic treatments ranges from 24 to 77% in root-filled teeth with periradicular lesions [8,9,10,11]. In some instances, *E. faecalis* has been the most dominant organism detected in pure cultures from root-filled teeth with periradicular lesions, emphasizing its potential role as a primary pathogen in endodontic failures. This association with various forms of periradicular disease, especially chronic asymptomatic periradicular lesions compared to symptomatic conditions, highlights the need for heightened vigilance and targeted therapeutic strategies [8]. Its remarkable resilience, partly attributed to its proton pumps that neutralize pH environments, has earned it the title of “survivor” of persistent infections [12].

The bacterium’s prevalence spikes in rinsed root canal samples from patients at various stages of endodontic treatment—those undergoing initial treatment, midway through treatment, and those receiving endodontic retreatment—compared to individuals with no history of endodontic treatments [13].

In light of these challenges, the role of chlorhexidine (CHX) in endodontic therapy has garnered significant attention. CHX is renowned for its potent antimicrobial properties, instrumental in eradicating microbial pathogens from the root canal system, though it is less effective than sodium hypochlorite (NaOCl) in biofilm disruption due to its limited ability to dissolve organic material [14]. Its cationic biocide nature enables it to breach microbial cell walls effectively, leading to the destruction of a wide array of bacteria and fungi, a critical step in curbing infections within the root canal [14]. Importantly, CHX’s use in clinical practice is widespread due to its broad-spectrum antimicrobial action, but its optimal use, especially at lower concentrations, remains underexplored in the context of biofilm-associated infections. CHX’s unique property of substantivity allows it to adhere to tissue surfaces, providing an extended antimicrobial effect that significantly bolsters disinfection efforts [15].

The literature continues to debate the optimal antiseptic solutions and their concentrations, although detailed data on CHX concentrations remain limited. Haut du formulaire Notably, some adverse effects have also been observed with the use of CHX at concentrations ranging from 0.2% to 2% [16].

Additionally, CHX’s efficacy is not limited to microbial eradication; it inhibits collagenolytic enzymes, thereby helping to preserve the structural integrity of periapical tissues during endodontic treatments [17]. These attributes underscore CHX’s indispensable role in the comprehensive cleaning and disinfection of the root canal, which is essential for the successful treatment of pulp and periradicular diseases. However, much of the literature focuses on higher concentrations of CHX, typically used in clinical settings. The potential benefits of using lower concentrations of CHX, such as reducing cytotoxicity to host tissues while still maintaining effective antimicrobial action, are less well documented and require further investigation.

The effectiveness of CHX against *E. faecalis*, often implicated in endodontic treatment failures, is particularly significant, because its ability to bind to hydroxyapatite and slowly release over time ensures a sustained antimicrobial presence in the root canal, offering extended protection against bacterial colonization [18,19]. This capability sets CHX apart from other disinfectants, which may dissipate more quickly, leaving the canal vulnerable to re-infection [20]. Despite these advantages, current protocols may overlook the effectiveness of CHX at sub-inhibitory concentrations, particularly in chronic or resistant infections, while understanding the exact mechanisms at lower concentrations could enhance the precision of antimicrobial strategies in endodontics, potentially offering a balance between efficacy and minimizing adverse effects.

The null hypothesis of this study is that low concentrations of CHX will have no significant antimicrobial or antibiofilm effects against *E. faecalis* when compared to untreated control samples. This hypothesis assumes that at sub-inhibitory concentrations, CHX will not cause notable disruption in bacterial growth, membrane integrity, biofilm formation, or gene expression.

The aim of this study is to evaluate the antimicrobial/antibiofilm mechanism action of low concentrations of CHX against *E. faecalis* within the context of endodontic treatment. Better understanding of the mode of action of CHX on *E. faecalis* will enable us to develop more effective endodontic treatment protocols, optimizing antimicrobial strategies to reduce the prevalence of treatment failures and enhance patient outcomes. Furthermore, by investigating the efficacy of CHX at lower concentrations, this study seeks to provide a more nuanced understanding of its potential as an auxiliary irrigant in the treatment of biofilm-associated infections.

This research aims to fill existing gaps in the literature regarding CHX’s mode of action when used at lower concentrations, and its broader implications for clinical endodontic protocols, with the hope of improving treatment efficacy and reducing persistent infections. Such an elaboration may pave way for safer root canal treatment.

## 2. Materials and Methods

### 2.1. Ethical Approval and PRILE 2021 Guidelines

The manuscript of this laboratory study has been written according to the Preferred Reporting Items for Laboratory studies in Endodontology (PRILE) 2021 guidelines (see Supplementary Materials, Figure S1).

This study was conducted with bacteria, and there was no need for ethical approval.

The PRILE 2021 guidelines are designed to ensure transparent and complete reporting of laboratory studies in endodontology, providing a structured approach to the methodology and findings.

Prile 2021: This study aimed to investigate the antibacterial mechanism of chlorhexidine (CHX) at varying concentrations against *Enterococcus faecalis* and its effectiveness in inhibiting biofilm formation, with the goal of improving endodontic treatment protocols. The research involved bacterial cultures, with no need for ethical approval, as only bacteria were used. *E. faecalis* ATCC 29212 was selected for the study, and different concentrations of CHX (0, 1.25, 2.5, 5, 10, and 20 μg/mL) were applied. Various growth media and staining agents were used for microscopy and flow cytometry analyses. Two experimental groups were included, with one group treated with CHX at varying concentrations and a control group with no CHX treatment. The dependent variables measured included bacterial growth inhibition, membrane permeability, membrane potential, biofilm formation, and gene expression changes. The methods employed included the use of a microplate reader for growth inhibition, flow cytometry for membrane assays, confocal and electron microscopy for structural assessments, and qRT-PCR for gene expression analysis. The study documented significant reductions in planktonic growth, colony-forming units (CFUs), membrane integrity disruption, altered biofilm structure, and changes in gene expression in response to CHX treatment. These findings highlight CHX’s potent antibacterial effects on *E. faecalis* through multiple mechanisms, supporting its use in endodontic disinfection.

### 2.2. Materials

Chlorhexidine di-gluconate (20%) (CHX) was purchased from Sigma Company (St. Louis, MO, USA).

*Enterococcus faecalis* (Andrewes and Horder) Schleifer and KilpperBalz (ATCC 29212) bacteria were obtained from the −80 °C stock of the Biofilm Research Laboratory. The initial bacterial culture was prepared by incubating 100 μL of the bacteria stock in 10 mL of brain–heart infusion broth (BHI; Acumedia, Lansing, MI, USA) for 16–18 h at 37 °C in a combined atmosphere of 95% air and 5% CO_2_.

Propidium iodide (PI) (Sigma, Livonia, MI, USA) was used for the membrane permeability assay as well as for the spinning disk confocal microscopy experiment. The cationic dye 3,3′-diethyloxacarbocyanine iodide (DiOC2(3); Molecular Probes, Eugene, OR, USA) was used for the membrane potential assay. Nile Red (APExBIO, Boston, MA, USA) and 4′,6-Diamidine-2′-phenylindole dihydrochloride (DAPI) (Sigma) were used for the Nile Red membrane staining assay. MTT solution (Calbiochem, Darmstadt, Germany) and dimethylsulfoxide (DMSO) (Bio-Lab Ltd., Jerusalem, Israel) were used for the high-resolution scanning electron microscopy (HR-SEM) experiment. SYTO 9 (Molecular Probes, Life Technologies, Carlsbad, CA, USA), and Alexa Fluor647-conjugated dextran 10,000 (Invitrogen, Thermo Fisher Scientific, Eugene, OR, USA) were used for the spinning disk confocal microscopy experiment. Gram’s crystal violet solution (Merck, EMD Millipore Corporation, Billerica, MA, USA) was used in an assay to stain the biofilm. For the quantitative real-time (qRT) polymerase chain reaction (PCR) analysis, we used RNA protect (Qiagen), Tri-Reagent (Sigma-Aldrich, St. Louis, MO, USA), a Fast Prep Cell Disrupter (Bio 101, Savant Instruments, Inc., New York, USA), chloroform (Bio-Lab, Jerusalem, Israel) and DNase- and RNase-free water (Promega, Fitchburg, WI, USA).

### 2.3. Controls and Sample Size Justification

Reference samples along with negative and positive controls were included in each set of experiments. The adequacy of the sample size was justified based on the power analysis to detect significant effects of CHX on *E. faecalis*, ensuring robust statistical evaluation, and depending on the different experiments’ characteristics.

The adequacy of the sample size was justified based on power analysis using a power of 80%, an effect size of 0.5, and a standard deviation derived from preliminary experiments. A sample size of n = 6 per concentration for CFU and n = 3 for other assays was determined to detect significant differences.

### 2.4. Inhibition of Planktonic Growth of E. faecalis

Overnight cultures of planktonic *E. faecalis* were incubated at 37 °C in an atmosphere of 95% air and 5% CO_2_ with BHI as a growth media, using a spectrophotometer at wavelength of 600 nm (A600 nm = 1.4) [21]. These cultures were then diluted in BHI to an OD_600nm_ = 0.1. Bacteria without any treatment acted as the control group. In this dose–response study, varying doses of CHX (ranging from 0 to 200 μg/mL at specific concentration intervals: 0, 0.0244, 0.0488, 0.0976, 0.1953, 0.3906, 0.7813, 1.5625, 3.125, 6.25, 12.5, 25, 50, 100, and 200 μg/mL) were administered to *E. faecalis* cultures in BHI. After 24 h of exposure, the absorbance values were recorded using a Tecan M200 microplate reader (Tecan Trading AG, Männedorf, Switzerland) maintained at 37 °C. This experiment was conducted in triplicate.

### 2.5. Colony Forming Units (CFU)

The colony forming units (CFU) assay was conducted at different incubation times (1, 2, 3, and 4 h) with CHX concentrations of 0, 5, 10, and 20 μg/mL. Serial dilutions were prepared in a tenfold manner by transferring 100 μL from each untreated and CHX-treated sample into another tube containing 900 μL of BHI, followed by thorough mixing. Subsequently, 100 μL of these bacterial mixtures were evenly spread onto BHI agar plates and then incubated overnight at 37 °C in a 5% CO_2_ atmosphere. After 24 h of incubation, colonies were enumerated utilizing the ImageJ software [22]. To calculate the original sample’s CFU per mL, the following formula was applied:

CFU per mL = (Number of colonies × dilution factor)/volume plated.

This experiment was conducted in sextuplicate.

### 2.6. Membrane Permeability Assay

The permeability of bacterial cell membranes was evaluated using PI, following the methodologies outlined by Cho and Ohsumi, in 2011 and 2015, respectively [23,24]. PI is capable of penetrating cells with compromised membranes, emitting red fluorescence upon binding to cellular nucleic acids [25]. Conversely, SYTO 9, which emits green fluorescence upon DNA binding, can passively enter cells, indicating its efficacy in marking both viable and non-viable cells due to its membrane permeability.

*E. faecalis* cultures, grown overnight, were diluted in BHI to an optical density (A_600nm_) equivalent to 0.3. These cultures were exposed to varying CHX doses (0, 1.25, 2.5, 5, 10, and 20 μg/mL) for 2 h at 37 °C. Subsequent to the treatment, the cells were stained with 10 μg/mL PI and 3.3 μM SYTO 9 for 20 min at 37 °C. Flow cytometry analysis was then conducted using a BD LSR-Fortessa flow cytometer (BD Biosciences, Erebodegem, Belgium), which was configured with five lasers (blue, red, violet, UV, and yellow–green), allowing for the optimization of assay design using the latest fluorescent dyes and substrates. The advantage of flow cytometry is that it analyzes individual bacteria, enabling the visualization of bacterial subpopulations.

A BD LSR-Fortessa was used to analyze the fluorescence signals from stained cells, with SYTO 9 detected through the AlexaFluor488 channel (blue laser excitation at 488 nm/emission filter at 530 nm) and PI through the dsRed channel (yellow–green laser excitation at 561 nm/emission filter at 586 nm), with data acquisition for green and red fluorescence, respectively. A total of 50,000 events were collected for each sample, performed in triplicates.

### 2.7. Membrane Potential (MP)

The membrane potential of *E. faecalis* was determined through the application of the DiOC2(3) utilizing flow cytometry, according to the protocol provided by the manufacturer, with modification by Chamlagain et al. [26]. DiOC2(3) is known for its green fluorescence in bacterial cells, with a shift to red fluorescence as the membrane potential increases [27]. Cultures of *E. faecalis* grown overnight were diluted in BHI to achieve an optical density (A_600nm_) equivalent to 0.3 and were then treated with varying levels of CHX (0, 1.25, 2.5, 5, 10, and 20 μg/mL) for 2 h at 37 °C, followed by incubation with 30 μM DiOC2(3) for 30 min at ambient temperature. Analysis of the samples was performed using an LSR-Fortessa flow cytometer (BD Biosciences), employing a 488 nm excitation laser and capturing emissions through green (530 nm) and red (620 nm) filters for data collection. The green-to-red fluorescence shift was analyzed cautiously, considering that while DiOC2(3) is a useful tool, it provides only indirect evidence of changes in membrane potential.

A total of 50,000 events were collected for each sample, performed in triplicates.

### 2.8. Nile Red Membrane Staining

Nile Red, a lipophilic stain, is utilized for its ability to selectively mark neutral lipids and lipophilic components within cells. It effortlessly integrates into areas rich in lipids and exhibits pronounced fluorescence when it binds to hydrophobic entities, such as lipid droplets. Control bacteria with an initial optical density (A_600nm_) equivalent to 0.3, as well as bacteria subjected to various concentrations of CHX (0, 1.25, 2.5, 5, 10, and 20 μg/mL) for 2 h at 37 °C, were stained with 10 μg/mL Nile Red and 1 μg/mL DAPI for 30 min at 37 °C, following the method described by Sugimoto et al. [28], or more recently carried out by Banerjee et al. [29]. The bacteria were then fixed in a 4% solution of glutaraldehyde for 40 min, and subsequently rinsed with double-distilled water. The prepared specimens were then placed onto mark neutral lipids and lipophilic components within cells. The stain effortlessly integrates into areas rich in lipids and exhibits pronounced fluorescence when it binds to hydrophobic entities. Subsequent to the staining process, the cells were rinsed with PBS and then subjected to flow cytometric analysis using an LSR-Fortessa flow cytometer (BD Biosciences). The analysis was carried out using a 561 nm yellow–green laser for excitation, with emission data collected through a 635 nm filter for Nile Red and a 405 UV laser for excitation, with emission data collected through a 450 nm filter for DAPI. This experiment was conducted in triplicate.

### 2.9. High Resolution Scanning Electron Microscopy (HR-SEM)

Planktonic *E. faecalis* with an initial OD_600nm_ of 0.3 underwent treatment with varying concentrations of CHX (0, 10 and 20 μg/mL) for a duration of 4 h. At the end of incubation, the bacterial samples were rinsed twice using phosphate-buffered glass slides, coated with iridium via sputtering, and examined under an Apreo 2 S (LoVac, Thermo Fisher Scientific, Waltham, MA, USA), as documented by Brandwein et al. [30]. The imaging process involved capturing photographs, at random, across four to five distinct regions. The analysis focused on counting the perforations present on the bacterial membrane in each image. For each treatment category, 200 bacteria were assessed based on 8 to 9 separate images taken at magnification of 20,000.

Perforation counting was conducted by manual identification using ImageJ software, based on visual inspection of 8 to 9 high-magnification (×20,000) images per treatment group. This method was validated by independent observers to ensure reproducibility.

### 2.10. Biofilm Analysis by Spinning Disk Confocal Microscopy (SDCM)

Spinning disk confocal microscopy (SDCM) was utilized to assess the architecture of the biofilm and to identify live/dead bacteria and extracellular polysaccharides (EPS) fol-lowing CHX treatment. Biofilms were cultivated on 8-well ibidi tissue culture-treated μ-slides either with or without varying CHX concentrations (0, 0.625, 1.25, 2.5, 5, 10, and 20 μg/mL) for 24 h, followed by two PBS washes. They were then stained with 3.3 µM SYTO 9, 10 µg/mL propidium iodide, and 10 µg/mL Alexa Fluor647-conjugated dextran 10,000 for 20 min at ambient temperature, where the Alexa Fluor647-conjugated dextran 10,000 stains the EPS. After staining, biofilms were rinsed with double-distilled water (DDW), fixed in 4% paraformaldehyde for 20 min, and preserved in 50% glycerol in DDW. Imaging was performed using a Nikon spinning disk confocal microscope (Nikon Corporation, Tokyo, Japan) with sections taken at 2.5 µm intervals, creating three-dimensional representations of bacterial and EPS distribution within the biofilms via the NIS-Element AR software. Analyses included three arbitrary fields per sample. Quantification of EPS production and the live/dead cell ratio were based on fluorescence intensity measurements using the NIS-Element AR software. The measurements were expressed as the ratio of EPS production to live and dead cells in each biofilm layer (2.5 µm intervals). The aggregate fluorescence intensity of EPS, live cells, and dead cells in CHX-treated biofilms was summed across all layers and compared to that of the untreated control, presenting a comprehensive evaluation of biofilm composition [31].

This experiment was conducted in triplicate.

### 2.11. Crystal Violet (CV) Staining of Biofilms

Biofilms were developed over a 24 h period on 96-well plates with CHX that was introduced while the biofilms were maturing, making this assay relevant for biofilm prevention rather than treatment. CHX concentrations of 0, 5, 10, and 20 μg/mL were used in these experiments. The biofilms were stained using 200 µL of 0.1% Crystal Violet (CV), which was prepared by diluting a 0.4% Gram’s crystal violet solution with double-distilled water (DDW), following the method previously described by Aqawi et al. [32]. After incubating for 15 min at room temperature, the excess of CV was discarded, and the wells were rinsed twice with DDW and left to dry overnight. To extract the CV stain, 150 µL of 33% acetic acid was added to each well, followed by shaking continuously for 5 min. The absorbance at 595 nm was then measured using the M200 Tecan plate reader (Tecan Trading AG, Männedorf, Switzerland) to assess the biofilm biomass. This experiment was conducted in triplicate.

### 2.12. Tetrazolium Reduction Assay (MTT Metabolic Assay)

This colorimetric assay is effective for assessing the metabolic activity of bacteria. As with the CV assay, biofilms were pre-formed prior to CHX treatment, making this assay clinically relevant for biofilm treatment.

The MTT metabolic assay was carried out following the protocol described before [32]. In summary, 50 µL of a 0.5 mg/mL MTT solution 253 (Calbiochem, Darmstadt, Germany) in PBS was applied to the biofilms in 96-well plates. The plates were then incubated for 1 h at 37 °C. After incubation, the wells were rinsed with PBS, and the tetrazolium precipitates from the biofilms were dissolved in 150 µL of dimethylsulfoxide (DMSO) (Bio-Lab Ltd., Jerusalem, Israel). Following a 10 min agitation on an orbital shaker, the absorbance at 570 nm was measured using the M200 Tecan plate reader. Here, n = 3 for each concentration. This experiment was conducted in triplicate.

### 2.13. Quantitative Real-Time (qRT)-Polymerase Chain Reaction (PCR) Analysis

The procedure was conducted in a manner consistent with the methodologies as described previously [26,33]. Planktonic bacteria were cultured in 15 mL tubes, with each sample comprising 5 mL of an overnight *E. faecalis* culture (OD = 0.2) mixed with 5 mL BHI broth containing 2.5 µg/mL CHX. Following a 2 h incubation, the bacterial cultures were centrifuged and then resuspended in 1 mL of RNA protect and kept on ice for 5 min. The RNA protect was removed by centrifugation, and the bacterial pellet were resuspended in 1 mL of Tri-Reagent, which was added to the bacterial pellets’ biofilms, before being washed and scraped into the Tri-Reagent using a sterile cell for total RNA extraction. The mixture was then transferred into 2 mL sterile screw-cap tubes filled with 200 µL of acid-washed glass beads for cellular disruption using a Fast Prep Cell Disrupter, for 45 s each time with strength 4.5, and then placed on ice 5 min; we repeated this process twice. After centrifuging to separate the glass beads, the supernatant was transferred to a new Eppendorf tube and mixed with 200 µL of chloroform, followed by vigorous mixing for 15 s. After a 15 min incubation at room temperature, the samples underwent centrifugation at 13,000 rpm for 15 min at 4 °C, with the upper phase (400 µL) being transferred to a new tube. Isopropanol (400 µL) was added to this phase for RNA precipitation, standing at room temperature for 30 min before centrifugation at 13,000 rpm for 30 min at 4 °C. The resultant RNA pellet was washed twice with 1 mL 75% ethanol, dried for 30 min at room temperature, and then dissolved in 20 μL ultrapure DNase- and RNase-free water. RNA purity and concentration were assessed by agarose gel electrophoresis and by using a Nanodrop ND-1000 Instrument (Wilmington, DE, USA). The RNA was then reverse transcribed to cDNA using the AB high-capacity cDNA reverse transcription kit (Applied Biosystems, Life Technologies, Waltham, MA, USA), followed by PCR amplification in a CFX96 BioRad Connect Real-Time PCR system with Luna Universal qPCR Master Mix (New England Biolabs GmbH) using 10 ng of cDNA and 300 nM of specific forward and reverse primers (Table 1). The PCR protocol included initial heating at 50 °C for 2 min, activation at 95 °C for 10 min, and 40 amplification cycles (95 °C for 15 s, 60 °C for 1 min). Gene expression analysis was performed using the 2^−ΔΔCt^ method with *16S rRNA*, *23S rRNA*, and *gyrA* and *gyrB* genes as the internal standards, normalizing the expression levels of treated samples to the controls. Three treated samples were calculated against each of three controls. Significant difference was determined when similar fold-change was observed for all treated samples when compared to all controls. This experiment was conducted in triplicate.

### 2.14. Statistical Analysis

The experiments were performed in triplicate and the data are presented as the average ± standard deviation. Statistical analyses were performed using Student’s *t*-test or ANOVA as appropriate, and differences were considered statistically significant when the *p*-value was less than 0.05.

## 3. Results

### 3.1. Anti-Bacterial Activity of CHX on E. faecalis

**Figure 1:**
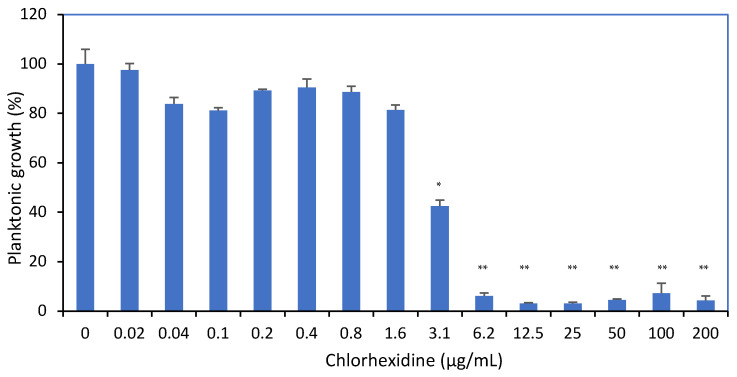
The effect of CHX on the planktonic growth of *E. faecalis*. Planktonic growth of *E. faecalis* treated with different concentrations of CHX for 24 h as measured by optical density (OD) at 600 nm. * *p* < 0.05; ** *p* < 0.01 compared with the control samples; n = 3.

Our initial aim was to determine the minimum inhibitory concentration (MIC) of CHX on the endodontic associated pathogenic bacteria *E. faecalis*. To this end, the bacteria were exposed to increasing concentrations of CHX for 24 h, and the turbidity was determined. CHX inhibited the growth of *E. faecalis* in a concentration-dependent fashion. A significant reduction in growth was observed at a concentration of 3.125 μg/mL CHX (58 ± 5% inhibition), reaching 94 ± 2% inhibition at 6.25 μg/mL (Figure 1—*p* < 0.01).

### 3.2. Using Colony Forming Units (CFU) to Assess the Initial Rate of the Antibacterial Effect of CHX on E. faecalis

**Figure 2:**
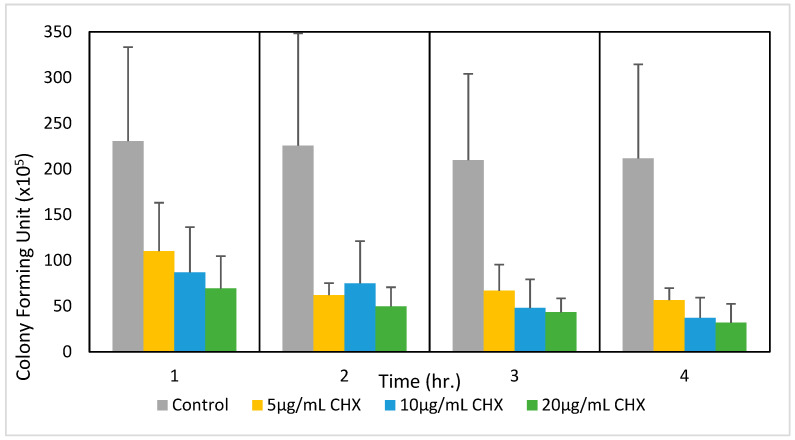
A time-course study of the effect of CHX on the colony-forming ability of *E. faecalis*. The bacteria at an initial OD_600nm_ of 0.3 were incubated in the absence or presence of the indicated concentrations of CHX for various time points, and the number of colony-forming units (CFUs) was determined by serial dilutions; n = 6.

To determine whether the effect of CHX on *E. faecalis* was bacteriostatic or bactericidal, the CFUs were counted at different time points after treating the bacteria with CHX at 0, 5, 10, and 20 μg/mL after various incubation times (1, 2, 3, and 4 h). Figure 2 shows a pronounced rapid suppression of bacterial proliferation in all CHX-treated samples, characterized by a concentration-dependent inhibition that persists up to 2 h of treatment. Beyond this period, the antibacterial effect is still present but reached a plateau. This observation suggests that CHX exhibits a maximal antibacterial action within the first 2 h of application. Consequently, it is inferred that a minimum exposure of 2 h to CHX is required to fully discern its antimicrobial effects on *E. faecalis*.

### 3.3. The Effect of CHX Treatment on Membrane Permeability

**Figure 3:**
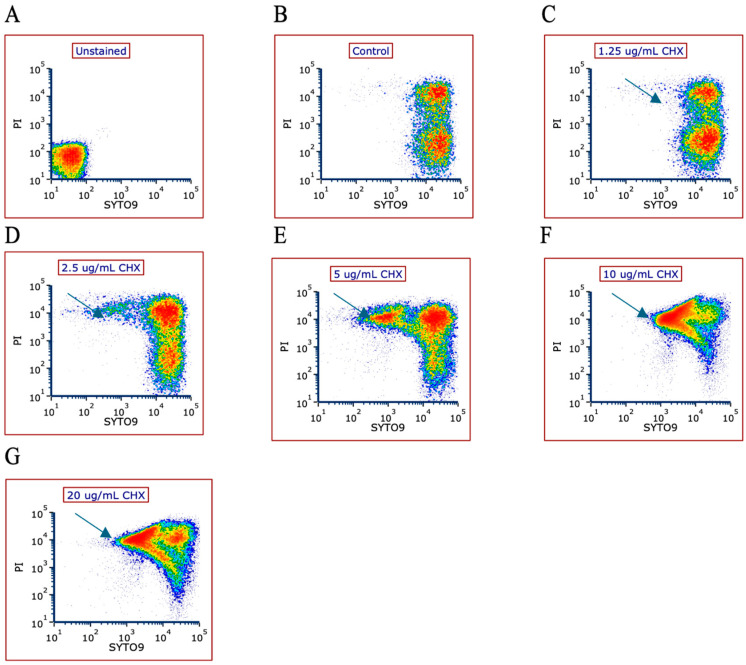

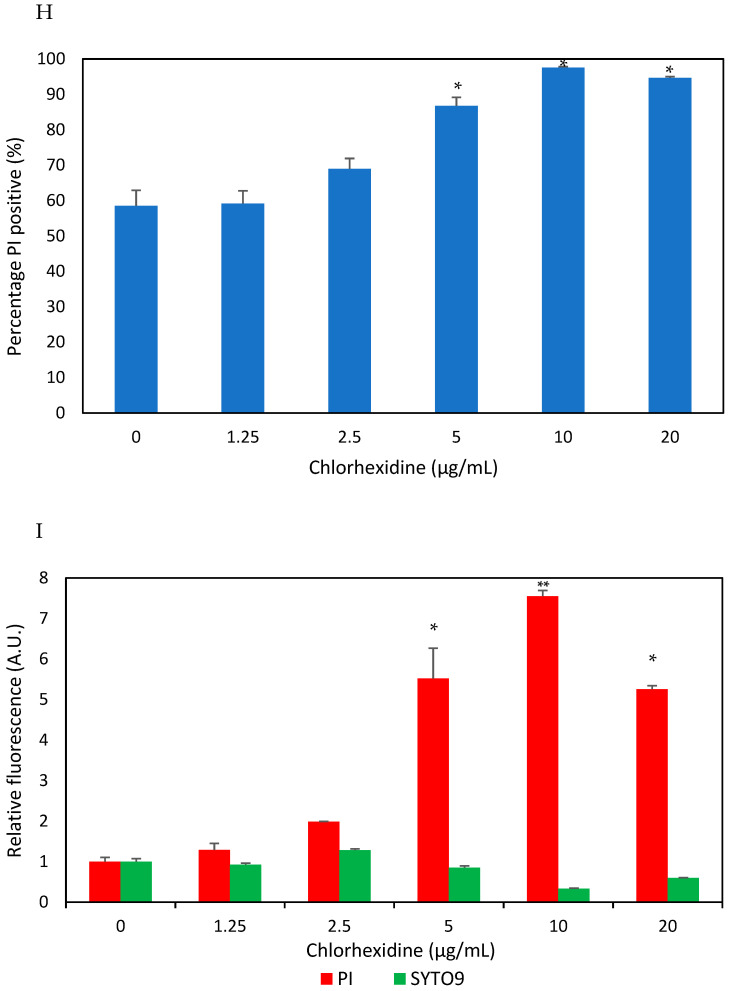
Increased membrane permeability following treatment with CHX. (**A**–**G**) PI versus SYTO 9 dot plot analysis of *E. faecalis* that was exposed to the indicated concentrations of CHX for 2 h. The blue arrows point to the SYTO 9 (low) and PI (high) bacterial populations, which represent bacteria with disrupted membranes. Red colors mean higher concentrations of bacteria. (**H**) The percentage of PI-positive bacteria. (**I**) The geometric mean fluorescence intensities of SYTO 9 (green) and PI (red). * *p* < 0.05 compared to control bacteria; ** *p* < 0.01 compared to control bacteria; n = 3.

Flow cytometric analysis of SYTO 9/PI-stained bacteria demonstrated that cells with disrupted membranes exhibited high PI and low SYTO 9 fluorescence, as evidenced in Figure 3D–G and summarized in Figure 3H. After a 2 h incubation period with CHX, an increase in the membrane permeability of *E. faecalis* was observed. The data plots reveal an escalating presence of bacteria with low SYTO 9 and high PI fluorescence at increasing CHX concentrations, indicating changes in the bacterial population. This trend is corroborated by Figure 3H, which shows a dose-dependent rise in the percentage of PI-high cells with increasing CHX concentrations. Additionally, a leakage of SYTO 9 from the bacteria was noted upon membrane compromise. The flow cytometry findings highlighted a decrease in SYTO 9 fluorescence intensity alongside an upsurge in PI fluorescence intensity following exposure to CHX concentrations of 2.5, 5, 10, and 20 µg/mL, as depicted in Figure 3I (*p* < 0.05).

This evidence collectively indicates that CHX treatment leads to bacterial membrane permeabilization in a concentration-dependent manner in *E. faecalis*.

### 3.4. CHX Induces Membrane Depolarization in E. faecalis

**Figure 4:**
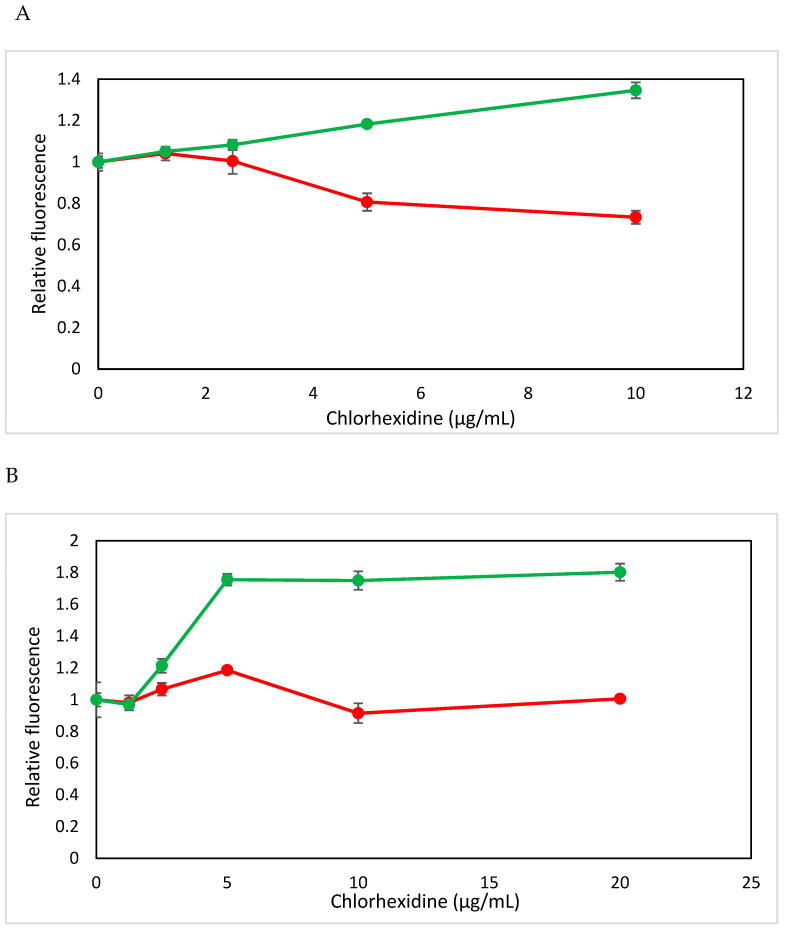
CHX causes an immediate reduction in membrane potential within 2 h. (**A**,**B**) The membrane potential was measured using the DiOC2(3) potentiometric dye during flow cytometry immediately after adding CHX and after 2 h treatment with CHX (**B**). Red fluorescence is an indication for the magnitude of the membrane potential. (**A**,**B**) are summaries of the relative fluorescence intensity (RFI) of the red (red lines) and green (green lines) fluorescence for the immediate CHX effect (**A**) and after 2 h treatment with CHX (**B**).

The membrane potential of *E. faecalis* was assessed both immediately after CHX addition and following a 2 h pre-treatment with CHX, using the potentiometric dye DiOC2(3). The introduction of CHX instigated an instant, dose-dependent diminution in red fluorescence intensity, coupled with an elevation in green fluorescence intensity. This shift signifies a prompt decline in membrane potential attributable to CHX exposure (Figure 4A). Subsequent to a 2 h incubation with CHX, the membrane potential of the bacteria remained notably diminished compared to the untreated control group (Figure 4B) and was even further reduced in comparison to the immediate CHX effect. It is very important to mention that the depolarization was negligible at the low CHX concentrations of 1.25 and 2.5 μg/mL but became significant at concentrations of 6.25 μg/mL CHX and higher. These findings underscore that CHX treatment leads to depolarization of the *E. faecalis* membrane potential, with the extent of depolarization intensifying over the duration of the 2 h treatment period.

### 3.5. The Effect of CHX Treatment on Nile Red Membrane Staining of E. faecalis

**Figure 5:**
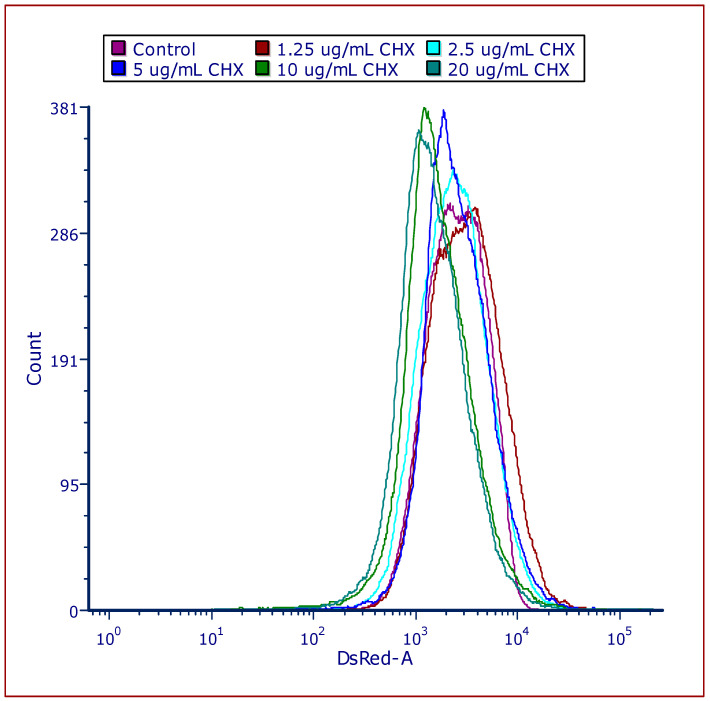
CHX reduced Nile Red staining of *E. faecalis* after a 2 h incubation. Flow cytometry of Nile Red fluorescence intensities of *E. faecalis* that was treated with the indicated concentrations of CHX for 2 h. The dose-dependent decrease in Nile Red staining highlights CHX’s ability to disrupt the bacterial membrane, particularly by altering the lipid composition. This membrane disruption leads to a decrease in the number of binding sites available for Nile Red, indicative of compromised membrane integrity, which is crucial for bacterial viability and resilience against antimicrobial agents.

The integration of Nile Red into bacterial membranes, which emits red fluorescence, serves as a method for quantifying changes in the bacterial membrane’s characteristics. Our observations indicate that CHX treatment led to a dose-dependent decrease in the staining following a 2 h incubation period (Figure 5). This trend is particularly evident with increasing concentrations of CHX, where the intensity of Nile Red staining diminished (as represented by transitions from black to green and then to light green shades), indicating a reduction in the availability or accessibility of lipid molecules within the bacterial membranes for binding by the dye. This trend suggests that CHX treatment alters the membrane’s lipid composition or structure, affecting its physical and functional properties. This reduction in staining intensity even at sub-minimum inhibitory concentration (sub-MIC) levels of CHX suggests that the compound’s impact is likely due to alterations in the membrane lipid content. This evidence points to the membrane-disruptive properties of CHX, further supporting its role in affecting *E. faecalis* viability by compromising membrane integrity.

### 3.6. CHX Creates Cell Pores and Affects the Morphology of E. faecalis

**Figure 6:**
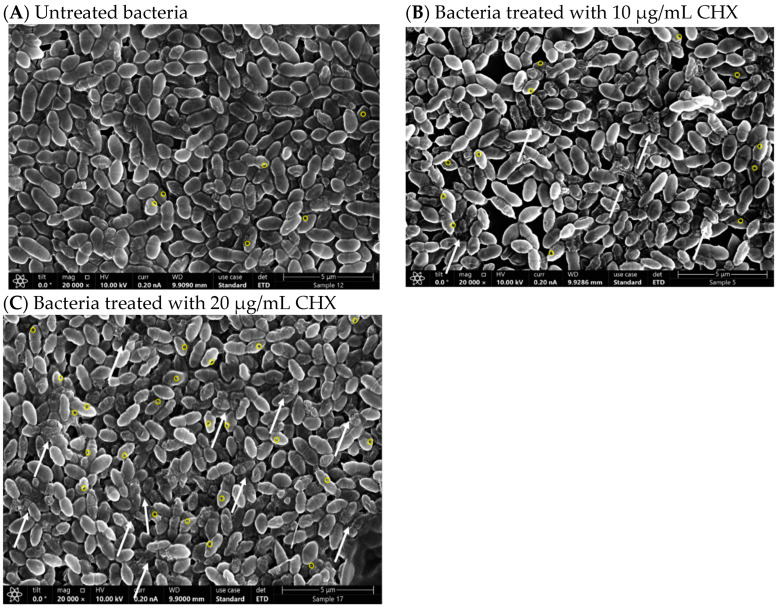

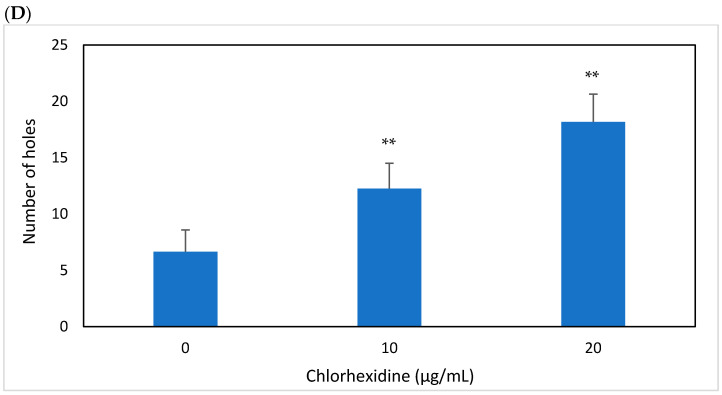
CHX alters the cell length and morphology of *E. faecalis*. (**A**) HR-SEM images (magnification ×20,000) of control bacteria; bacteria treated with 10 µg/mL CHX (**B**) and bacteria treated with 20 µg/mL CHX (**C**) for 2 h in planktonic growth conditions. The white arrows point to dysmorphic, swollen bacteria. (**D**) The number of pores in the bacteria’s membranes of the growing bacteria in the control, 10, and 20 µg/mL CHX-treated samples (yellow circles). ** *p* < 0.005 compared to control bacteria; n = 120–220.

**Figure 7:**
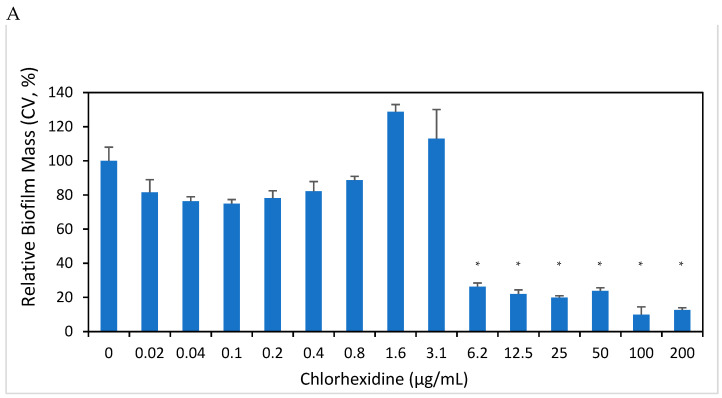

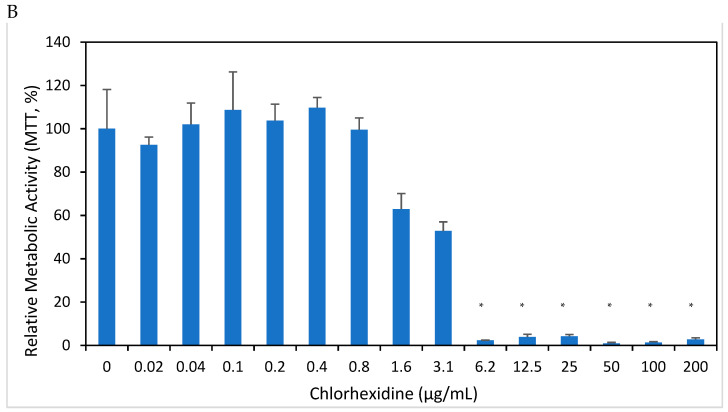
Anti-biofilm activity of CHX on forming biofilm on *E. faecalis*. (**A**) Biofilm mass of *E. faecalis* that have been incubated in the absence or presence of various CHX concentrations or respective ethanol concentrations for 24 h, as determined by crystal violet staining. n = 3; * *p* < 0.05. (**B**) Metabolic activity as measured by the MTT reduction for the biofilm from untreated and CHX-treated *E. faecalis*. n = 3; * *p* < 0.05.

To elucidate the impact of CHX on cellular morphology, *E. faecalis* cultures were subjected to varying concentrations of CHX and subsequently examined via high-resolution scanning electron microscopy (HR-SEM). Samples of planktonically grown bacteria were treated with CHX for a duration of 2 h (Figure 6A,B). Notably, bacteria exposed to 10 µg/mL CHX for 2 h exhibited dysmorphia and shrinking, as highlighted by white arrows in Figure 7B, which is in stark contrast to the untreated control samples depicted in Figure 6A. Moreover, bacteria subjected to a higher concentration of 20 µg/mL CHX for the same period showed even more pronounced dysmorphia and swelling, as denoted by the white arrows in Figure 6C.

The CHX treatment was also associated with the formation of numerous pores across the bacterial membranes, as indicated by the yellow circles in Figure 6B,C. Quantitative analysis of the pores, based on counting their occurrence in each HR-SEM image, revealed that control bacteria displayed an average of 6.64 ± 1.94 pores per image. In contrast, samples treated with 10 µg/mL CHX exhibited a significantly higher average of 12.26 ± 2.24 pores per image, and those treated with 20 µg/mL CHX demonstrated an even greater average of 18.16 ± 2.48 pores per image (Figure 6D—*p* < 0.005).

These observations from the HR-SEM images demonstrate that increasing concentrations of CHX induce progressively significant alterations in the morphology of *E. faecalis*. Such morphological changes are characterized by the development of membrane pores (marked in yellow) and an increasing prevalence of bacterial cells that appear smaller and dysmorphic.

### 3.7. CHX Reduces E. faecalis Biofilm, as Well as the Metabolic Activity of Forming Biofilms

We tested the effect of CHX on the forming biofilm of *E. faecalis*. CV staining shows that CHX at concentrations equal to and higher than 6.25 µg/mL reduced the biofilm mass of *E. faecalis* by more than 95% (Figure 7A; *p* < 0.05). Additionally, the metabolic activity of the biofilms was strongly reduced at concentrations equal to and higher than 6.25 µg/mL CHX (75% reduction) (Figure 7B; *p* < 0.05).

### 3.8. Reduction in Biofilm Viability and Extracellular Polymeric Substance Production by E. faecalis Treated with CHX

**Figure 8:**
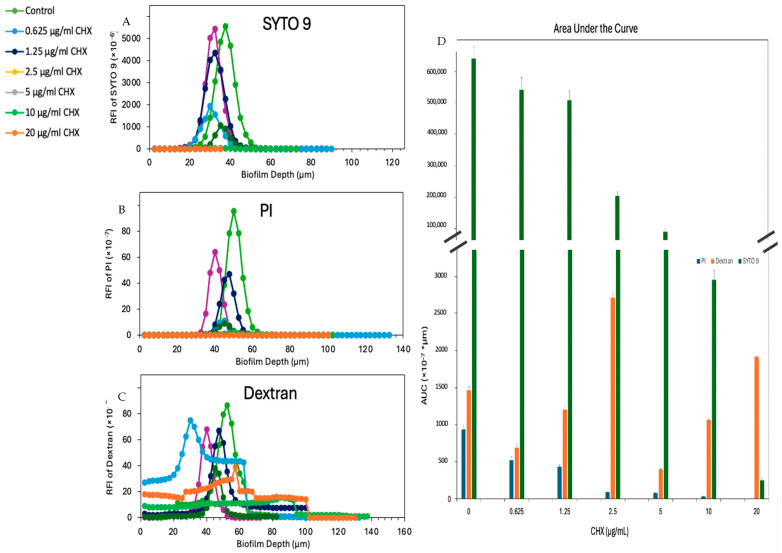
CHX reduces the biofilm mass as well as the EPS production of *E. faecalis* after a 24 h incubation in BHI supplemented with 2% sucrose. (**A**–**C**) The relative fluorescence intensities (RFI) of SYTO 9 (**A**), PI (**B**), and dextran (**C**) in each biofilm layer captured at intervals of 2.5 µm (after *E. faecalis* biofilms were incubated in the absence or presence of various concentrations of CHX and 0.05% ethanol for 24 h and then stained with Alexafluor647-conjugated anionic dextran 10,000, SYTO 9 and PI for 30 min, and the images were captured by a spinning disk confocal microscope). (**D**) The AUC of the RFI graphs of SYTO 9 (green), PI (blue), and dextran (orange); n = 3.

To explore the impact of CHX on the production of the extracellular polymeric substance (EPS) matrix—a key component facilitating biofilm formation and bacterial adhesion—biofilms of *E. faecalis*, both untreated and treated with CHX, were visualized using spinning disk confocal microscopy (SDCM) after undergoing live/dead and EPS staining. No alteration in SYTO 9 fluorescence was observed at a CHX concentration of 0.625 µg/mL (Figure 8A). However, at higher CHX concentrations, a pronounced dose-dependent decrease in SYTO 9 staining was noted (Figure 8A), aligning with previously mentioned findings indicating a substantial reduction in viable immobilized bacteria at these concentrations (Figure 3). Concurrently, PI staining was also diminished at these higher concentrations (Figure 8B), correlating with the notable decrease in overall bacterial counts within the biofilms. Significantly, the EPS production, as indicated by the intensity of fluorescently labeled dextran 10,000 staining, exhibited marked reductions in line with increasing CHX concentrations (Figure 8C). This decline in EPS intensity parallels the reduced bacterial population, suggesting that CHX not only affects the viability of bacteria within biofilms but also impairs their ability to produce EPS, thereby potentially diminishing the structural integrity and protective capabilities of the biofilm matrix. From the AUC calculations (Figure 8D), we can confirm our hypothesis, as we note a decrease in the PI and SYTO 9 values of the AUC with the increasing concentrations of CHX. For the dextran staining, we can observe a relative increase in EPS production below the MIC in Figure 8D, and, from 5 µg/mL CHX, a relative decrease in EPS production is recorded. It is important to note that it is not a dose-dependent decrease.

### 3.9. CHX Reduces the Expression of Various Genes Involved in Biosynthesis of Virulence Factors and Other Binding Proteins of E. faecalis

**Figure 9:**
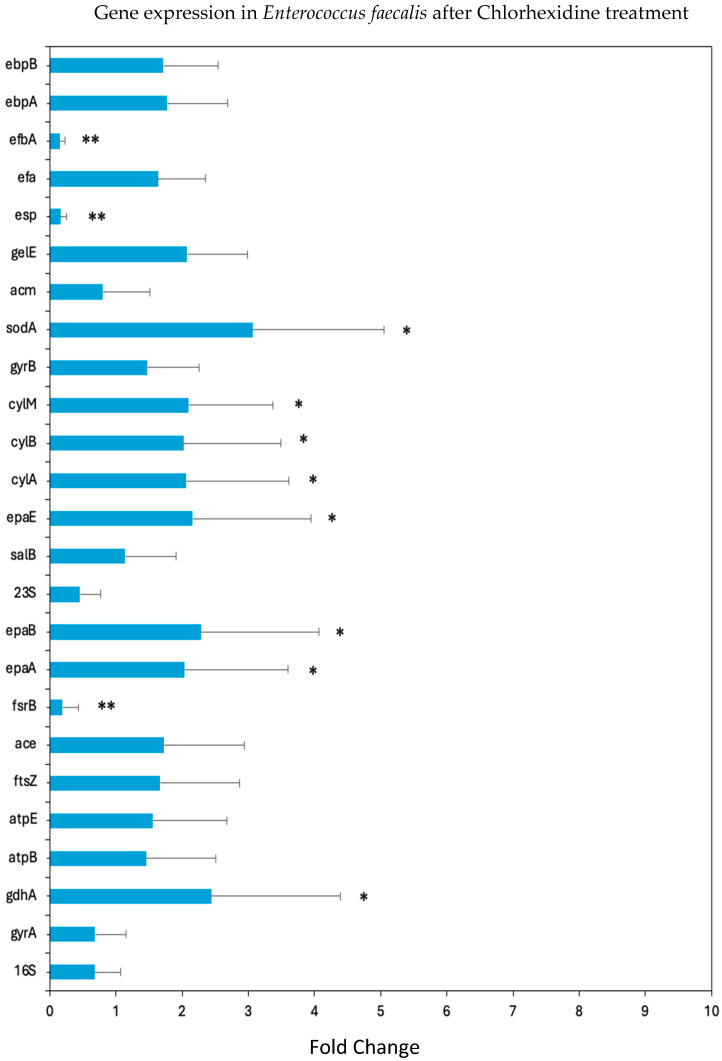
Effect of CHX on gene expression in *E. faecalis*. Real-time PCR analysis of various genes after a 2 h treatment of *E. faecalis* with 2.5 μg/mL CHX; n = 3. The relative expression levels of the genes analyzed by real-time PCR were normalized against *16S rRNA*, *23S rRNA*, and *gyrA* and *gyrB*, which served as internal standards. * *p* < 0.05; ** *p* < 0.01 compared to *16S rRNA*, *23S rRNA*, and *gyrA* and *gyrB* genes.

Our data (Figure 9) show several changes in the gene expression of *E. faecalis* pattern after a 2 h incubation in the presence of 2.5 µg/mL CHX. There was a significant downregulation of the *fsrB* gene involved in the Fsr quorum-sensing system in *E. faecalis* (*p* < 0.01). Significant downregulation was also observed in other genes related to biofilm formation including: *esp* and *efbA* (*p* < 0.01). On the contrary, there was a slight increase in the expression of the *epaA*, *epaB*, and *epaE* genes involved in the biosynthesis of cell wall polysaccharides (*p* < 0.05), that contribute to the virulence and survival of *E. faecalis* in the host environment [34]. There was also an increase in the gene expression of *soda* (*p* < 0.05), which encodes for superoxide dismutase (SOD). Superoxide dismutase is an important antioxidant enzyme that plays a critical role in protecting the bacterium from oxidative stress by catalyzing the dismutation of superoxide radicals into molecular oxygen and hydrogen peroxide [35]. Also, the *cylA*, *cylB*, and *cylM* genes, which are involved in the virulent cytolysin production, were upregulated (*p* < 0.05).

## 4. Discussion

This study investigated the mechanistic mode of action of low concentrations of CHX against *E. faecalis*, a resilient pathogen commonly associated with endodontic treatment failures. This bacterium is known for its resilience to traditional endodontic disinfectants and its ability to penetrate deeply into dentinal tubules, which makes it difficult to eradicate completely during routine treatments [5]. CHX, as a broad-spectrum antiseptic agent, is frequently used in dental medication and is considered one of the preferred agents for treating intracanal infections [17]. While CHX’s antimicrobial properties are well-known, this study sought to deepen the understanding of its specific action on biofilm formation and biofilm maturation and mode of action of its antibacterial activity at lower concentrations than those typically used in dental clinics. These findings provide important insights for refining its clinical application. This new data is important for refining its clinical application in root canal infections and improving treatment success rates.

The significant reduction in planktonic growth and CFUs of *E. faecalis* at minimum inhibitory concentrations (MIC) of 6.25 µg/mL CHX highlights the compound’s potent antibacterial effect. This result is in line with previous studies that document the susceptibility of Gram-positive bacteria, including *E. faecalis*, to CHX’s [36,37]. The concentration-dependent inhibition of bacterial growth emphasizes the importance of dosing in clinical applications to optimize therapeutic outcomes. In comparison, sodium hypochlorite (NaOCl), a commonly used agent in endodontics, has an MIC of around 3600 μg/mL for *E. faecalis*, while calcium hydroxide (Ca(OH)_2_) has an MIC of around 750 μg/mL depending on the strain and testing methodology [38,39]. This means that higher concentrations of NaOCl and Ca(OH)_2_ than CHX are required to inhibit the growth of *E. faecalis*. The killing kinetics also plays a significant role in the antibacterial properties of any antibacterial agent: a dose-dependent effect of CHX was found on *E. faecalis*, along the scientific line showing that its antibacterial effects increase as the exposure time to different bacteria increases [40].

The mode of action of low CHX concentrations on *E. faecalis* was examined, its impact on membrane integrity, depolarization and permeability provide mechanistic insights into its bactericidal activity. The increased membrane permeability observed by flow cytometry through propidium iodide (PI) uptake suggests that CHX disrupts *E. faecalis* cell walls, enabling PI entry and SYTO 9 cytoplasmic leakage, which are indicative of compromised cell viability. This mechanism reflects CHX’s ability to target and destabilize bacterial membranes, a key aspect of its bactericidal action [41]. The subsequent observations of membrane depolarization and reduced Nile Red staining further substantiate the hypothesis that CHX alters membrane potential and lipid compositions, which are critical factors in bacterial survival and virulence. In comparison, NaOCl’s mode of action involves oxidative properties that disrupt cell membranes and cause cell lysis through protein denaturation and nucleic acid degradation [42]. Both agents have an effect on the bacteria’s membrane, but these effects are of different natures.

The morphological changes in *E. faecalis* induced by CHX at low concentrations, as evidenced by HR-SEM, including the formation of pores and shrinking of bacterial cells, underscore the physical disruptions caused by CHX exposure on the cells. These alterations, particularly the pore formation, are indicative of CHX’s destabilizing effects on cell membranes, which likely contribute to its bactericidal efficacy. This physical disruption complements the observed biochemical and cellular changes, painting a comprehensive picture of CHX’s multifaceted antibacterial action. Interestingly, similar morphological changes have been observed in studies on NaOCl’s effect on *Candida albicans* using SEM imaging [43].

The field of endodontics therapy relies heavily on the efficacy of antimicrobial agents to manage infections within the complex anatomy of root canals. The unique challenges of intracanal treatments include the eradication of biofilms and deeply entrenched bacteria in the microscopic tubular structure of dentine. CHX’s role in endodontics, therefore, is not only about its antimicrobial properties but also its ability to affect biofilm, penetrate matured biofilms, and maintain a bactericidal environment over extended periods, crucial for the success of both primary and retreatment endodontic therapies. The findings from this study support the potential refinement of CHX clinical protocols in endodontic treatments to enhance microbial eradication and prevent reinfection, a common complication in endodontic failures.

We have also examined the effect of low concentrations of CHX on forming biofilm. Affecting forming biofilm requires a different mode of action than treating matured biofilm. It usually requires high concentrations of an agent to disrupt an existing biofilm. CHX reduced both biofilm mass and EPS production, suggesting that it can penetrate biofilms of *E. faecalis*, affect bacteria vitality and disrupt their structural integrity by probably inhibiting the synthesis if commonly found ESPs, such as glucans and fructans [44].

At the molecular level, the study provides novel insights into CHX’s influence on gene expression in *E. faecalis*, and showcases how CHX not only exhibits direct antibacterial properties but also intricately influences the pathogenic behavior of *E. faecalis* by modulating key gene expressions critical to its survival and virulence within the host. A notable finding is the significant downregulation of the *fsrB* gene, pivotal in the Fsr quorum-sensing system of *E. faecalis* [45]. This downregulation suggests a CHX-induced disruption in the bacterial communication systems vital for coordinating pathogenic activities, including biofilm formation. The concurrent strong downregulation of the genes *esp* and *efbA*, both of which are closely associated with *E. faecalis*’s ability to form robust biofilms, further exemplifies CHX’s multifaceted inhibitory effects on mechanisms underlying bacterial aggregation, protection, and persistence. Contrastingly, the slight increase in the expression of genes, such as *epaA*, *epaB*, and *epaE*, which are central to the biosynthesis of cell wall polysaccharides, particularly EPA [46], suggests an adaptive response of *E. faecalis* to maintain its cell wall integrity and virulence under CHX assault. This adaptive mechanism, however, may also spotlight potential therapeutic targets for undermining *E. faecalis*’s resilience and pathogenicity. Similarly, the upregulation of the *sodA* gene, encoding the superoxide dismutase (SOD) enzyme [47], underscores the bacterium’s countermeasures against oxidative stress, likely induced by CHX. This enzyme plays a crucial role in detoxifying harmful superoxide radicals, suggesting that *E. faecalis* may leverage this upregulation in a bid to survive the oxidative stress conditions fostered by CHX treatment.

Gene expression analysis has revealed a notable upregulation of virulence-associated genes, including *cylA*, *cylB*, and *cylM*, which are involved in cytolysin production [46], suggesting a complex interaction between CHX exposure and the modulation of *E. faecalis* virulence, where the bacterium may enhance its virulence as a countermeasure to external stressors.

The effect of an agent on gene expression may give many indications as far as its mode of action is concerned. Interestingly, in terms of the upregulation of genes associated with virulence, cytolysin production presents a novel insight into the antibacterial action of CHX against *E. faecalis*. The suppression of these genes not only highlights CHX’s direct bactericidal activity but also suggests its role in undermining the pathogenic potential of *E. faecalis* by targeting mechanisms essential for its colonization and virulence. This dual action—eliminating bacteria while reducing their virulence—is especially important in endodontic treatments, where both infection eradication and the prevention of recurrence are critical [48].

As CHX is one of the suggested active agents in intracanal medication, the primary aim of this study was to evaluate the efficacy of CHX against *E. faecalis* with the aim of improving endodontic treatment outcomes. Compelling evidence underscoring CHX’s potential as an antibacterial agent is a critical adjunct in endodontic disinfection protocols. The findings bolster the proposition that CHX, particularly in formulations that ensure sustained release within the root canal system, could significantly improve the management of endodontic infections, reducing treatment failure rates associated with *E. faecalis* infections.

In light of these findings, it is evident that CHX represents a potent antimicrobial agent with clear applicability in endodontic protocols. The study’s implications extend to the development of advanced therapeutic strategies, which could usher in a new paradigm in the prevention of post-treatment infections. This study’s implications extend to developing advanced therapeutic strategies that could enhance root canal disinfection, addressing the challenge of bacterial persistence and reinfection. These insights enrich our understanding of CHX’s role in endodontic treatments, emphasizing its capacity to influence not just bacterial survival but also pathogenic attributes critical to infection persistence and recurrence.

One of the major strengths of this study is its comprehensive approach to understanding the antibacterial mechanisms of CHX at relative low concentrations. The use of multiple assays to assess different aspects of bacterial viability, such as planktonic growth, biofilm formation/maturation, gene expression, and the structural integrity of bacterial cell membranes, allowed for a multifaceted evaluation of CHX’s efficacy. High-resolution imaging and advanced molecular biology methods provided detailed insights into the cellular and molecular changes induced by CHX treatment, while the statistical analysis ensures that the findings are robust and provide a reliable basis for further research and clinical applications.

The findings that CHX is active at low concentrations is important, as the pharmacokinetics of any drug application vary in terms of concentration in the target organ. The clinical dosage of CHX in the root canal can reach up to 2%, and its profile may show low values at given time. The range within which CHX affected *E. faecalis* in this study was 100–1000 times less than the usual dosage of CHX, and the findings here may suggest that the use of lower concentrations of CHX is sufficient to cause an antibacterial/anti-biofilm effect. This finding widens the therapeutic window for CHX in root canal treatment.

The limitations of this study—particularly the in vitro nature of the experiments and the focus on mono-species biofilms—suggest that future research should incorporate multi-species biofilms, including clinical isolate species and in vivo studies to validate these findings. Moreover, exploring synergies between CHX and other agents could improve biofilm eradication and endodontic treatment outcomes.

Future research should focus on optimizing the delivery and formulation of CHX within the endodontic space to maximize its antibacterial efficacy. Additionally, exploring synergies between CHX and other antimicrobial agents could offer new strategies for overcoming resistance and enhancing the disinfection of the root canal system.

## 5. Conclusions

- Effectiveness of CHX: The study studies new aspects of CHX as a potent antibacterial agent against *E. faecalis*, effectively disrupting cell membranes, inducing morphological changes, affecting gene expression, and inhibiting biofilm formation and mature biofilm.
- Rationale and Impact: Supported by the experimental data, the findings highlight CHX’s crucial role in enhancing endodontic treatment outcomes by significantly reducing bacterial load and affecting bacterial viability.
- Main Takeaway: CHX remains a valuable antiseptic in endodontics, essential for improving the effectiveness of treatment protocols and potentially reducing the incidence of treatment failures.

## Figures and Tables

**Table 1:** Primers used for real-time PCR.

Gene	Forward Primer	Reverse Primer
*16S rRNA*	CCGAGTGCTTGCACTCAATTGG	CTCTTATGCCATGCGGCATAAAC
*23S rRNA*	CCTATCGGCCTCGGCTTAG	AGCGAAAGACAGGTGAGAATCC
*gyrA*	CGGATGAACGAATTGGGTGTGA	AATTTTACTCATACGTGCTT
*cylA*	CAAGTTGCTGGAGTAATAGACACGAT	TCCCATCCATCACCTTGTAAGAA
*cylB*	CATGGTACACAAGTTGCTGGAGTAA	CCCATCCATCACCTTGTAAGAATT
*cylM*	GTATTTAGAATCACTAGGATTCTTTGTAGGAA	GGAATTTCAGAATCTAGGTTTCTCAATAA
*epaA*	TTGCATCACCGCTTGTTATC	TCGCCAACTAGACCGATTAG
*epaB*	CGGATACAGAAACAACGGAT	TAGAGAATCCGATAGCCTGC
*epaE*	CGAAGTCAAATTACGCAGT	AGGATTCGTGTGTGCCTGTA
*acm*	GGCCAGAAACGTAACCGATA	AACCAGAAGCTGGCTTTGTC
*ace*	GGCGACTCAACGTTTGAC	TCCAGCCAAATCGCCTAC

## Data Availability

The raw data supporting the conclusions of this article will be made available by the authors on request.

## Supplementary Materials

The following supporting information can be downloaded at: https://www.mdpi.com/article/10.3390/microorganisms12112297/s1, Figure S1: PRILE 2021 Flowchart. Reference [49] is cited in the supplementary materials.

## References

[1] Kakehashi S., Stanley H.R., Fitzgerald R.J. (1965). The effects of surgical exposures of dental pulps in germ-free and conventional laboratory rats. Oral Surg. Oral Med. Oral Pathol. Oral Radiol. Endod..

[2] Peters O.A., Schönenberger K., Laib A. (2001). Effects of four Ni-Ti preparation techniques on root canal geometry assessed by micro computed tomography. Int. Endod. J..

[3] Zhu W.C., Gyamfi J., Niu L.N., Schoeffel G.J., Liu S.Y., Santarcangelo F., Khan S., Tay K.C., Pashley D.H., Tay F.R. (2013). Anatomy of sodium hypochlorite accidents involving facial ecchymosis—A review. J. Dent..

[4] Flemming H.C., Wingender J., Szewzyk U., Steinberg P., Rice S.A., Kjelleberg S. (2016). Biofilms: An emergent form of bacterial life. Nat. Rev. Microbiol..

[5] Distel J.W., Hatton J.F., Gillespie M.J. (2002). Biofilm formation in medicated root canals. J. Endod..

[6] Gomes B.P., Ferraz C.C., Vianna M.E., Berber V.B., Teixeira F.B., Souza-Filho F.J. (2001). In vitro antimicrobial activity of several concentrations of sodium hypochlorite and chlorhexidine gluconate in the elimination of *Enterococcus faecalis*. Int. Endod. J..

[7] Saatchi M., Shokraneh A., Navaei H., Maracy M.R., Shojaei H. (2014). Antibacterial effect of calcium hydroxide combined with chlorhexidine on *Enterococcus faecalis*: A systematic review and meta-analysis. J. Appl. Oral Sci..

[8] Rôças I.N., Siqueira J.F., Santos K.R. (2004). Association of *Enterococcus faecalis* with different forms of periradicular diseases. J. Endod..

[9] Fouad A.F., Zerella J., Barry J., Spångberg L.S. (2005). Molecular detection of *Enterococcus* species in root canals of therapy-resistant endodontic infections. Oral Surg. Oral Med. Oral Pathol. Oral Radiol. Endod..

[10] Baumgartner J.C., Siqueira J.F., Xia T., Róças I.N. (2004). Geographical differences in bacteria detected in endodontic infections using polymerase chain reaction. J. Endod..

[11] Sundqvist G., Figdor D., Persson S., Sjögren U. (1998). Microbiologic analysis of teeth with failed endodontic treatment and the outcome of conservative re-treatment. Oral Surg. Oral Med. Oral Pathol. Oral Radiol. Endod..

[12] Portenier I., Waltimo T., Haapasalo M. (2003). *Enterococcus faecalis*—The Root Canal Survivor and ‘Star’ in Post-Treatment Disease. Endod. Top..

[13] Sedgley C.M., Lennan S.L., Clewell D.B. (2004). Prevalence, phenotype and genotype of oral enterococci. Oral Microbiol. Immunol..

[14] Mohammadi Z., Abbott P.V. (2009). The properties and applications of chlorhexidine in endodontics. Int. Endod. J..

[15] Raheja J., Tewari S., Tewari S., Duhan J. (2014). Evaluation of efficacy of chlorhexidine intracanal medicament on the periodontal healing of concomitant endodontic-periodontal lesions without communication: An interventional study. J. Periodontol..

[16] Alves F.R.F., Marceliano-Alves M.F., Souza A.C., Campello A.F. (2020). Mucosal Fenestration After 2% Chlorhexidine Extrusion Used in Substitution of Sodium Hypochlorite: A Case Report. Eur. J. Dent..

[17] Gomes B.P., Vianna M.E., Zaia A.A., Almeida J.F., Souza-Filho F.J., Ferraz C.C. (2013). Chlorhexidine in endodontics. Braz. Dent. J..

[18] Molander A., Reit C., Dahlén G., Kvist T. (1998). Microbiological status of root-filled teeth with apical periodontitis. Int. Endod. J..

[19] Rölla G., Löe H., Schiott C.R. (1970). The affinity of chlorhexidine for hydroxyapatite and salivary mucins. J. Periodont. Res..

[20] Messer H.H., Chen R.S. (1984). The duration of effectiveness of root canal medicaments. J. Endod..

[21] Steinberg D., Moreinos D., Featherstone J., Shemesh M., Feuerstein O. (2008). Genetic and physiological effects of noncoherent visible light combined with hydrogen peroxide *on Streptococcus mutans* in biofilm. Antimicrob. Agents Chem..

[22] Rodrigues P.M., Luís J., Tavaria F.K. (2022). Image Analysis Semi-Automatic System for Colony-Forming-Unit Counting. Bioengineering.

[23] Cho J., Lee D.G. (2011). The characteristic region of arenicin-1 involved with a bacterial membrane targeting mechanism. Biochem. Biophys. Res. Commun..

[24] Ohsumi T., Takenaka S., Wakamatsu R., Sakaue Y., Narisawa N., Senpuku H., Ohshima H., Terao Y., Okiji T. (2015). Residual structure of *Streptococcus mutans* biofilm following complete disinfection favors secondary bacterial adhesion and biofilm re-development. PLoS ONE.

[25] Veerman E.C., Nazmi K., Van’t Hof W., Bolscher J.G., Den Hertog A.L., Nieuw Amerongen A.V. (2004). Reactive oxygen species play no role in the candidacidal activity of the salivary antimicrobial peptide histatin 5. Biochem. J..

[26] Chamlagain M., Hu J., Sionov R.V., Steinberg D. (2024). Anti-bacterial and anti-biofilm activities of arachidonic acid against the cariogenic bacterium *Streptococcus mutans*. Front. Microbiol..

[27] Lin W., Fan S., Liao K., Huang Y., Cong Y., Zhang J., Jin H., Zhao Y., Ruan Y., Lu H. (2023). Engineering zinc oxide hybrid selenium nanoparticles for synergetic anti-tuberculosis treatment by combining *Mycobacterium tuberculosis* killings and host cell immunological inhibition. Front. Cell. Infect. Microbiol..

[28] Sugimoto A., Maeda A., Itto K., Arimoto H. (2017). Deciphering the mode of action of cell wall-inhibiting antibiotics using metabolic labeling of growing peptidoglycan in *Streptococcus pyogenes*. Sci. Rep..

[29] Banerjee S., Sionov R.V., Feldman M., Smoum R., Mechoulam R., Steinberg D. (2021). Anandamide alters the membrane properties, halts the cell division and prevents drug efflux in multidrug resistant *Staphylococcus aureus*. Sci. Rep..

[30] Brandwein M., Al-Quntar A., Goldberg H., Mosheyev G., Goffer M., Marin-Iniesta F., López-Gómez A., Steinberg D. (2016). Mitigation of Biofilm Formation on Corrugated Cardboard Fresh Produce Packaging Surfaces Using a Novel Thiazolidinedione Derivative Integrated in Acrylic Emulsion Polymers. Front. Microbiol..

[31] Aqawi M., Steinberg D., Feuerstein O., Friedman M., Gingichashvili S. (2022). Cannabigerol Effect on *Streptococcus mutans* Biofilms—A Computational Approach to Confocal Image Analysis. Front. Microbiol..

[32] Aqawi M., Sionov R.V., Gallily R., Friedman M., Steinberg D. (2021). Anti-Biofilm Activity of Cannabigerol against *Streptococcus mutans*. Microorganisms.

[33] Feldman M., Ginsburg I., Al-Quntar A., Steinberg D. (2016). Thiazolidinedione-8 Alters Symbiotic Relationship in *C. albicans*-*S. mutans* Dual Species Biofilm. Front. Microbiol..

[34] Ramos Y., Sansone S., Morales D.K. (2021). Sugarcoating it: Enterococcal polysaccharides as key modulators of host-pathogen interactions. PloS Pathog..

[35] Zheng M., Liu Y., Zhang G., Yang Z., Xu W., Chen Q. (2023). The Applications and Mechanisms of Superoxide Dismutase in Medicine, Food, and Cosmetics. Antioxidants.

[36] Pereira A.P., Antunes P., Willems R., Corander J., Coque T.M., Peixe L., Freitas A.R., Novais C. (2022). Evolution of Chlorhexidine Susceptibility and of the EfrEF Operon among *Enterococcus faecalis* from Diverse Environments, Clones, and Time Spans. Microbiol. Spectr..

[37] Kim H.S., Woo Chang S., Baek S.H., Han S.H., Lee Y., Zhu Q., Kum K.Y. (2013). Antimicrobial effect of alexidine and chlorhexidine against *Enterococcus faecalis* infection. Int. J. Oral Sci..

[38] Ganesh Kumar A., Joseph B., Nandagopal S., Sankarganesh P., Jagdish S.K. (2019). Experimental Human Root Canal Irrigant NaOCl Against *Enterococcus faecalis* and 3T3, and Determination of Cytotoxicity Effect. Biomed. Pharmacol. J..

[39] Putri C.M.D., Diani P., Malinda Y. (2022). The MIC and MBC of calcium hydroxide medicament against bacteria that cause chronic periapical abscess in the vulnerable initial 7-days of endodontic treatment. Padj. J. Dent..

[40] Vianna M.E., Gomes B.P., Berber V.B., Zaia A.A., Ferraz C.C., de Souza-Filho F.J. (2004). In vitro evaluation of the antimicrobial activity of chlorhexidine and sodium hypochlorite. Oral Surg. Oral Med. Oral Pathol. Oral Radiol. Endod..

[41] Rzycki M., Drabik D., Szostak-Paluch K., Hanus-Lorenz B., Kraszewski S. (2021). Unraveling the mechanism of octenidine and chlorhexidine on membranes: Does electrostatics matter?. Biophys. J..

[42] Eriksson S., van der Plas M.J.A., Mörgelin M., Sonesson A. (2017). Antibacterial and antibiofilm effects of sodium hypochlorite against *Staphylococcus aureus* isolates derived from patients with atopic dermatitis. Br. J. Dermatol..

[43] da Silva P.M., Acosta E.J., Pinto Lde R., Graeff M., Spolidorio D.M., Almeida R.S., Porto V.C. (2011). Microscopical analysis of *Candida albicans* biofilms on heat-polymerised acrylic resin after chlorhexidine gluconate and sodium hypochlorite treatments. Mycoses.

[44] Gränicher K.A., Karygianni L., Attin T., Thurnheer T. (2021). Low Concentrations of Chlorhexidine Inhibit the Formation and Structural Integrity of Enzyme-Treated Multispecies Oral Biofilms. Front. Microbiol..

[45] Ran S., He Z., Liang J. (2013). Survival of *Enterococcus faecalis* during alkaline stress: Changes in morphology, ultrastructure, physiochemical properties of the cell wall and specific gene transcripts. Arch. Oral Biol..

[46] Liu F., Sun Z., Wang F., Liu Y., Zhu Y., Du L., Wang D., Xu W. (2020). Inhibition of biofilm formation and exopolysaccharide synthesis of *Enterococcus faecalis* by phenyllactic acid. Food. Microbiol..

[47] Lata P., Ram S., Shanker R. (2016). Multiplex PCR based genotypic characterization of pathogenic vancomycin resistant *Enterococcus faecalis* recovered from an Indian river along a city landscape. Springerplus.

[48] Rathnayake I.U., Hargreaves M., Huygens F. (2012). Antibiotic resistance and virulence traits in clinical and environmental *Enterococcus faecalis* and *Enterococcus faecium* isolates. Syst. Appl. Microbiol..

[49] Nagendrababu V., Murray P.E., Ordinola-Zapata R., Peters O.A., Rôças I.N., Siqueira J.F., Priya E., Jayaraman J., Pulikkotil S.J., Camilleri J. (2021). PRILE 2021 guidelines for reporting laboratory studies in Endodontology: A consensus-based development. Int. Endod. J..

